# Trans-ethnic study design approaches for fine-mapping

**DOI:** 10.1038/ejhg.2016.1

**Published:** 2016-02-03

**Authors:** Jennifer L Asimit, Konstantinos Hatzikotoulas, Mark McCarthy, Andrew P Morris, Eleftheria Zeggini

**Affiliations:** 1 Wellcome Trust Sanger Institute, Cambridge, UK; 2 Wellcome Trust Centre for Human Genetics, University of Oxford, Oxford, UK; 3 Department of Biostatistics, University of Liverpool, Liverpool, UK

## Abstract

Studies that traverse ancestrally diverse populations may increase power to detect novel loci and improve fine-mapping resolution of causal variants by leveraging linkage disequilibrium differences between ethnic groups. The inclusion of African ancestry samples may yield further improvements because of low linkage disequilibrium and high genetic heterogeneity. We investigate the fine-mapping resolution of trans-ethnic fixed-effects meta-analysis for five type II diabetes loci, under various settings of ancestral composition (European, East Asian, African), allelic heterogeneity, and causal variant minor allele frequency. In particular, three settings of ancestral composition were compared: (1) single ancestry (European), (2) moderate ancestral diversity (European and East Asian), and (3) high ancestral diversity (European, East Asian, and African). Our simulations suggest that the European/Asian and European ancestry-only meta-analyses consistently attain similar fine-mapping resolution. The inclusion of African ancestry samples in the meta-analysis leads to a marked improvement in fine-mapping resolution.

## Introduction

Numerous genome-wide association studies (GWASs) have been carried out, resulting in the identification of many susceptibility loci for a wide range of complex traits.^1^ The detection of additional loci has resulted from GWAS meta-analyses (primarily in populations of European descent) and has been aided by imputation that allows the prediction of genotypes not typed on GWAS chips, but present in a higher density reference. Nonetheless, the joint effects of the loci identified to date have only accounted for a small proportion of the heritability of complex traits. Because of linkage disequilibrium (LD), many variants within identified loci have indistinguishable signals. This LD is beneficial to GWAS, as it increases the power to detect new associations, when the causal variant is not directly typed. However, the caveat to this is that it limits the potential of fine-mapping efforts to refine the location of causal variants.

GWAS data from non-European populations are increasing in availability, and this provides the opportunity to meta-analyse GWAS across ancestrally diverse populations. Trans-ethnic meta-analysis may lead to an increase in power to detect novel loci and may improve fine-mapping resolution of causal variants by leveraging differences in the structure of LD between diverse populations.^2, 3^ The inclusion of African ancestry samples may yield substantial improvements in localisation of causal variants because of low LD and high genetic heterogeneity.^4^

Several trans-ethnic analyses have shown empirical improvements in fine-mapping resolution. Examples include a refinement of signals at several type II diabetes loci in an analysis involving samples of European, East Asian, South Asian, and Mexican and Mexican-American ancestry,^5^ as well as in previously identified adiposity loci in a trans-ethnic meta-analysis of samples from European and African ancestries.^6^ Similarly, a refinement of signals was obtained at several lipid trait loci in a trans-ethnic fine-mapping study involving African-American, East Asian, and European ancestries.^7^ However, a comparison of study designs, where the ancestral composition of the samples varies in diversity whereas the same number of samples (and cost) is constant, has not yet been performed. Approaches to meta-analysis include a fixed- or random-effects model, as implemented in GWAMA (genome-wide association meta-analysis^8^) or MANTRA (Meta-ANalysis of Trans-ethnic Association studies), that allows for heterogeneity in allelic effects between distantly related populations.^9^ We focussed on fixed-effects meta-analysis, as trans-ethnic studies of complex traits demonstrated relatively little evidence of heterogeneity in allelic effects at common variant loci. Moreover, MANTRA and fixed-effects GWAMA have been shown to have similar power when the variant has a non-null effect in all of the populations.^10^ Therefore, our objective is to examine different study designs with respect to ancestral composition, rather than statistical approaches to meta-analysis. There may be power and fine-mapping advantages in increasing the ancestral diversity among the samples while retaining the same number of samples and cost.

We carried out an extensive simulation study to examine different study designs with respect to ancestral composition (European, East Asian, and African) to assess any power and fine-mapping advantages due to increasing ancestral diversity among samples while retaining the same number of samples and cost. The aim is to determine whether trans-ethnic meta-analyses offer fine-mapping resolution advantages over single ancestry meta-analyses, and whether or not any gains are due to the inclusion of African ancestry samples.

## Materials and methods

We focus on five established type II diabetes (T2D) loci (*IGF2BP2*, *CDKN2A/B*, *KCNQ1*, *FTO*, *CDKAL1*). These loci were explicitly chosen as they have association signals present in multiple ethnic groups,^11^ and several of these loci have shown large differences in LD between ethnic groups (eg, *CDKAL1* and *KCNQ1*). Such loci characteristics are likely to favour success for trans-ethnic meta-analyses.

In this simulation study, trans-ethnic meta-analysis was carried out by a frequentist fixed-effects meta-analysis that was implemented in GWAMA^8^ as a proof of principle in comparing fine-mapping of studies having varying degrees of ancestral diversity. In addition, for completeness, we also employed GWAMA using random effects in a selection of settings. Fine-mapping assessment was examined when the variant had a non-null effect in all of the populations and under various levels of ancestral diversity: European-only samples; moderate ancestral diversity (European and Asian samples); and high ancestral diversity (European, East Asian, and African samples).

### Simulation settings

Sets of six cohorts were meta-analysed across five loci under various allelic heterogeneity models and ancestry compositions of the contributing cohorts. Data were simulated using Hapgen2^12^ from 6 populations based on the 1000 Genomes June 2011 haplotypes:^13^ CEU, TSI (European reference panels (RPs)), CHB, JPT (East Asian RPs), LWK, and YRI (African RPs). Each cohort was composed of 1000 cases/1000 controls, and 1000 replications were used for each setting. These simulations represent directly typed data, and variants are referred to as perfect. They were then thinned down to GWAS density based on the SNPs in the Illumina 660-Quad array, and subsequently imputed via IMPUTE2,^14^ using the same cross-population 1000 Genomes reference panel, and effective population size Ne=20 000. A 500 kb up- and down-stream buffer was included in the imputation, and variants with SNPTEST proper information score below 0.4 were filtered out. Both perfect (all variants are directly typed) and imputed data were analysed. Analysis of the perfect data illustrates an optimal scenario and provides the maximum possible power and refined fine-mapping resolution that may be attained, whereas the imputed data represent a more realistic setting.

There were three general ancestry combinations considered for meta-analysis:

1. Single ancestry (European): 6 CEU samples;
2. Moderate ancestral diversity (European and Asian): 3 CEU+TSI samples, 3 CHB+JPT samples; and
3. High ancestral diversity (European, Asian, and African): 1 sample from each of CEU, TSI, CHB, JPT, LWK, and YRI.

Within each locus, causal variants were selected within ±0.5% of one of three minor allele frequencies (MAFs): 5, 10, or 20%, with respective relative risks of 1.4, 1.3, and 1.2. As the MAF varies between the populations, when there was a shared causal variant c_1_, it was selected to satisfy the MAF requirements in the CEU population frequency and to be nonmonomorphic in the other populations. A single causal variant c_1_ was considered for each of the single ancestry and moderate and high ancestral diversity scenarios.

### Meta-analysis and fine-mapping

Within each study sample, single SNP association tests were carried out with an additive model in a ‘missing data likelihood' framework, as implemented in SNPTEST v2.^15^ As put into practice in GWAMA,^8^ the frequentist fixed-effects meta-analysis of the single SNP summary statistics assumes that the allelic effect at SNP *j* is the same across all samples. This meta-analysis approach is highly computationally efficient, but has the limitation that it assumes a homogeneous effect size among all of the samples.

A measure of fine-mapping resolution was based on the construction of 95% credible sets using Bayesian theory, such that the sets were 95% likely to encompass the causal SNP. In order to form the credible sets, the association summary statistics (B̂ and its variance, *V*) from each SNP were first converted to an approximate Bayes' factor (ABF):


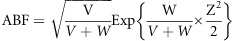


where 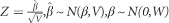 and *N(μ,σ*^*2*^) denotes that the random variable follows a Normal distribution with mean *μ* and variance *σ*^*2*^.^16^ For a case–control study and an additive model, a widely used default value is *W=0.04*.^15^ Evidence against the null hypothesis increases with the ABF value, and posterior probabilities were calculated from the ratio of the individual ABF value to the sum of the ABF values for all SNPs within the region. These posterior probabilities were then ranked in decreasing order and SNPs were consecutively collected to form a 95% credible set until the total of the posterior probabilities for the SNPs in the set first exceeded 0.95.^17^

We only constructed 95% credible sets when the lead SNP had a *P*-value below a prespecified threshold in order to avoid the construction of sets consisting only of SNPs that are unlikely to be disease associated. The construction of credible sets based on lead SNPs with weak signals requires a larger number of SNPs to meet the criterion of a certain total posterior probability, as the individual SNP posterior probabilities tend to be small in this case. For computational efficiency, we focussed on a frequentist threshold for the decision of whether or not to construct a credible set. This avoided an additional computational step of ABF calculations that would not be required if it was inappropriate to construct a credible set.

In assessing any effect of ancestral diversity on fine-mapping resolution, the probability of the causal variant being a member of the 95% credible set was assessed among simulation scenarios in which all samples shared a causal variant. For each region, the median number of SNPs within credible sets that contained the true causal variant was considered in evaluating the refinement for localising the causal variant within the locus; a smaller number is indicative of higher fine-mapping resolution. As a means of combining the measures over the regions, we refer to the average of the region-specific medians as a summary statistic.

Effects of allelic heterogeneity were investigated by introducing a different causal variant c_2_ for the Asian samples, such that it was of similar MAF, but in low LD (*r*^2^<0.2) with the European causal variant. In particular, for the moderate ancestral diversity scenario, we compared the setting of no heterogeneity, where c_1_ is the causal variant for both European and East Asian samples, with the allelic heterogeneity setting of causal variant c_1_ for European samples and causal variant c_2_ for East Asian samples.

To reflect locus heterogeneity, in the high ancestral diversity set-up we assumed null associations at both c_1_ and c_2_ for the African samples; we refer to such cohorts as null. In these heterogeneity scenarios we examined the impact on power to detect the variant that is causal in the European samples. We compared the settings of no heterogeneity (causal variant c_1_ in all samples), allelic and locus heterogeneity (causal variants c_1_ in European samples and c_2_ for East Asian samples; null for African samples), and locus heterogeneity (causal variant c_1_ in European samples; null for East Asian and African samples). For both types of heterogeneity, comparisons are also made with the power to detect a causal variant in the single ancestry (European) composition to assess the power loss of trans-ethnic meta-analyses in the presence of heterogeneity.

## Results

In the decision for construction of 95% credible sets, a stringent lead SNP *P*-value threshold of 5 × 10^−8^ was employed. The probability that the 95% credible set contained the true causal variant was high across all five loci and there was no clear collective pattern between probabilities and degree of ancestral diversity. Upon collapsing the individual region results into averages over the regions, any differences were smoothed over and there were negligible differences between the probabilities for the various diversity settings at a given causal variant MAF (Table 1). For the high diversity scenario with perfect data, a random-effects meta-analysis was also implemented using GWAMA. The summary measure over the five regions of the probability that the 95% credible set contains the causal variant drops slightly with random effects, compared with fixed effects, and the standard error of these estimates are slightly higher for random effects. The respective summary probabilities for MAFs 5, 10, and 20% are 0.911, 0.900, and 0.912 (with standard errors 0.00437, 0.00453, and 0.00489) for random effects, whereas the coinciding probabilities for fixed effects are 0.930, 0.910, and 0.928 (with standard errors 0.00395, 0.00421, and 0.00440).

**Figure 1:**
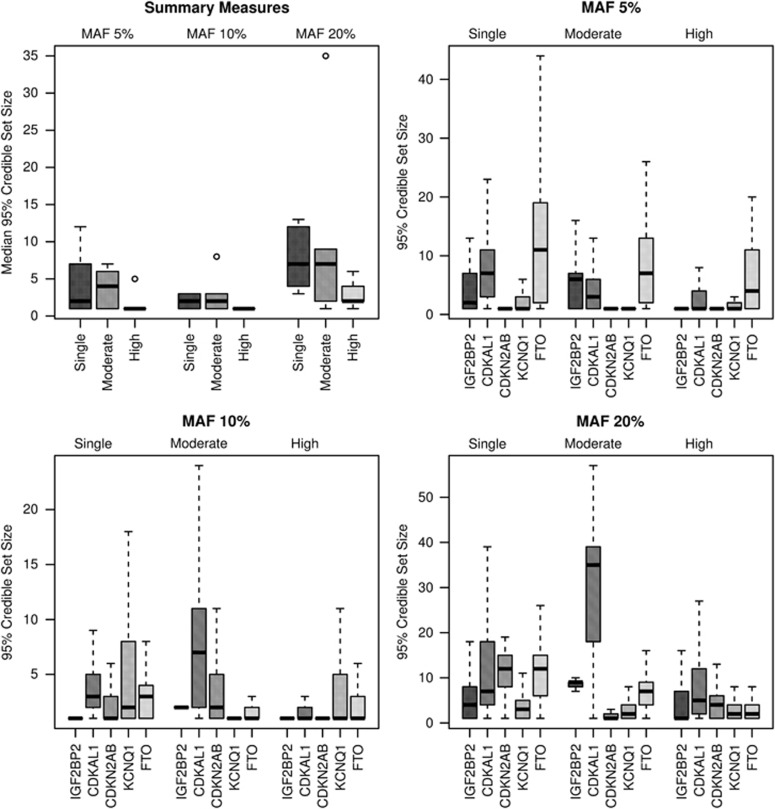
Boxplots, for perfect data, of the number of SNPs in the 95% credible sets for summary measures of the five loci and for individual loci at MAFs 5, 10, and 20% and ancestral diversities single, moderate, and high.

**Figure 2:**
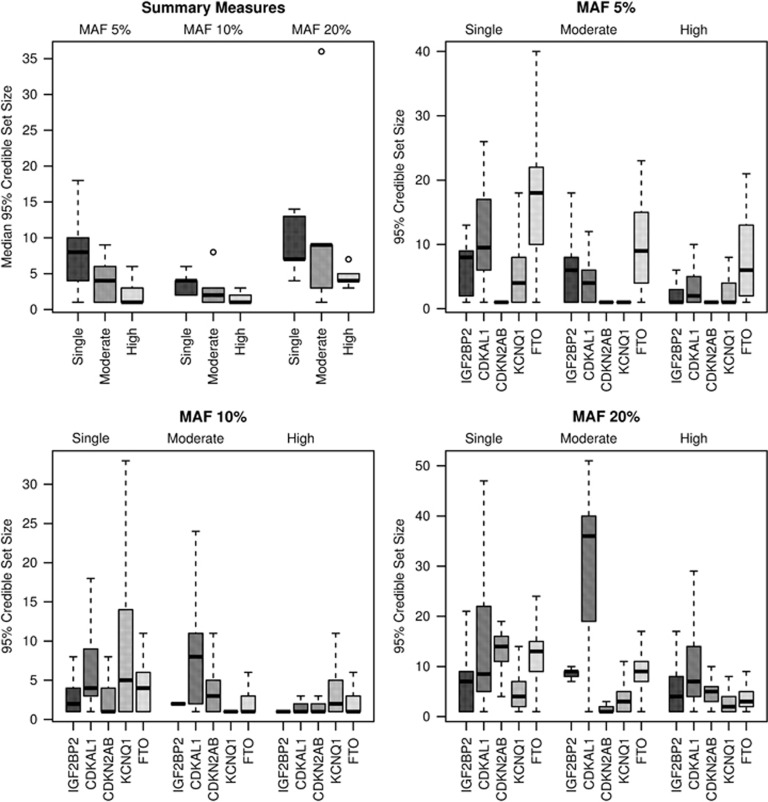
Boxplots, for imputed data, of the number of SNPs in the 95% credible sets for summary measures of the five loci and for individual loci at MAFs 5, 10, and 20% and ancestral diversities single, moderate, and high.

Next, we examined the median number of SNPs in the credible sets for each region. Summary measures over the regions are provided in Table 2, and individual region results are available in Supplementary Table S1. Boxplots displaying the distribution of the number of SNPs in 95% credible sets are given for summary measures and individual loci in Figures 1 and 2 for perfect and imputed data, respectively. That is, each summary measure boxplot gives the distribution of the median number of SNPs in the 95% credible sets constructed for each locus, whereas the individual loci boxplots display the distributions for the number of SNPs in the 95% credible sets. The summary statistics suggested that the high ancestral diversity meta-analysis had the smallest median number of SNPs in 95% credible sets, regardless of causal variant MAF and whether or not the data were imputed; at MAF 10%, for increasing levels of diversity, the perfect data summary medians were 2, 3, and 1, whereas for imputed data they were 3.6, 3, and 1.6 (Table 2). In most of the individual region analyses, the highly diverse ancestral composition meta-analysis achieved the smallest median or the same median as the moderate ancestral diversity meta-analysis (Supplementary Table S1). In the random-effects meta-analyses of the high diversity setting for perfect data, the summary measures over the five loci for the median number of SNPs in the 95% credible sets were 1.6, 1.0, and 2.8 at MAFs 5, 10, and 20%, respectively; the credible sets constructed from the random-effects meta-analyses are similar to those for fixed effects. The locus-level results are not presented for this random-effects (RE) analysis, as they are identical to the fixed-effects (FE) analysis, except for smaller medians at MAF 20% for *CDKAL1* (RE 5 SNPs; FE 6 SNPs) and at MAF 5% for *FTO* (RE 4 SNPs; FE 5 SNPs).

Within each region we used the Kruskal–Wallis rank sum test to assess the evidence for the null hypothesis that the median was the same in each ancestral setting group against the alternative that at least one group had a different median. If there was evidence of a difference, Dunn's test was then applied to test each of the pairwise comparisons for the null hypothesis that, with probability 0.5, a random SNP count from the first group is larger than one from the second group. This may be interpreted as a test for one group having a larger median than the other.

The Kruskal–Wallis test, applied to perfect data, resulted in strong evidence (*P*-value <10^−5^) that at least one ancestral setting had a median SNP count different from the others, with the exception of one region and MAF setting. Similarly high levels of significance (*P*-value <10^−5^) were attained for all imputed data settings.

In both perfect and imputed data, for each region with a causal variant having MAF 10% or 20%, Dunn's test gave strong evidence (*P*-value <10^−5^) of the high ancestral diversity group having a lower median than the single ancestry group. This was also true at the lower MAF for each region, except for only moderate evidence at *CDKN2A/B* (perfect and imputed data *P*-values 0.0302 and 0.0973). There was not a clear trend between the European and European/Asian meta-analyses, as either group was found to have strong support (*P*-value <10^−5^) for a larger median or no evidence (*P*-value >0.1) for a difference in medians for various MAFs and regions.

As each gene may differ in LD structure, we paired the medians of each ancestral diversity comparison according to gene, and then tested whether the region medians of lower ancestral diversity settings had a greater average than those of higher ancestral diversity via a paired *t*-test. Detailed results are given in Supplementary Table S2. For perfect data, over all MAF settings, there was moderate evidence (considering the small sample size of 5 regions) that the regional median numbers of SNPs in credible sets were on average larger in European-only meta-analyses than in those that include African and East Asian ancestry samples (*P*-values 0.071, 0.045, and 0.035 for MAFs 5, 10, and 20%, respectively). Imputed data had similar levels of evidence (*P*-values 0.023 and 0.047 for MAFs 5 and 20%, respectively) and considering 0.05 Bonferroni-corrected threshold of *α*=0.011 for the three pair-wise comparisons, this holds with strong evidence for MAF 10% (*P*-value 5.5E-03). The results also suggested that the European/Asian trans-ethnic meta-analysis did not have a greater fine-mapping refinement than the European-only meta-analysis, except for moderate evidence in the low MAF setting of imputed data (*P*-value 0.032). There was moderate evidence for a higher median in European/Asian compared with European/Asian/African at MAF 5% (*P*-values 0.051 and 0.069 for perfect and imputed data, respectively).

As we found a clear improvement in the trans-ethnic meta-analyses composed of European, East Asian, and African populations, but not in those without African samples, we also investigated fine-mapping in African-only meta-analyses of perfect data. Based on the same simulation setting of 1000 cases/1000 controls for each sample and the same causal variants (based on European populations), where three samples are taken from each of the LWK and YRI populations, we found that the proportion of 95% credible sets that contain the causal variant is similarly high for the various ancestral diversity settings. However, the standard errors of these probabilities are slightly higher for the African-only samples, as there are fewer replications in which the lead SNP passes the *P*-value threshold of 5 × 10^−8^ (Table 1). In addition, the median tends to be lowest in the African-only meta-analyses (see Table 2 for summaries and Supplementary Table S1 for individual loci).

Fine-mapping resolution is most refined for African-only meta-analyses, suggesting that features of the African populations such as lower LD rather than ethnic diversity are the underlying source of improvement in the high diversity settings. The allele spectrum between the populations was also examined; for the various European causal variant MAF settings, the median MAFs for the East Asian and African samples are provided in Supplementary Table S3. The median MAF in the African samples tends to be larger than that of the European samples, whereas the median MAF in the East Asian samples tends to be similar or larger in comparison with European samples. However, there is no clear pattern related to MAF and fine-mapping improvement, indicating that this improvement is more related to LD.

We also examined the effects of ancestral diversity on power to detect an association with a variant that was causal in all samples. At significance level 0.05, regardless of the level of ancestral diversity among the samples, a power close to or identically 100% was attained for both perfect and imputed data, respectively. Upon repeating the meta-analysis with random effects for *IGF2BP2* in the high ancestral diversity setting and for the three MAF settings, identical power results of 1.0 were attained for perfect data.

For imputed data, the causal variant may either be a tag SNP, so that it is directly typed, or it may be imputed. Among the replications for imputed data, upon examination of the power only at the imputed causal variants, there are not substantial differences compared with the power at directly typed variants. For instance, when the causal variant is the same across all ancestries and diversity settings, the power remains close to 1.0 at imputed-only causal variants, except that at MAF 5% the power is slightly lower for single ancestry in *FTO* (4% lower) and in *CDKN2AB* (8% lower). In the presence of locus or allelic heterogeneity, there are also very few differences in power between imputed and directly typed causal variants; at MAF 5%, there is occasionally a power reduction near 10% when causal variants are imputed rather than directly typed. As the differences are not substantial, this subset of results from the imputed data is not provided.

The effect of allelic heterogeneity on the power to detect a causal variant was assessed by comparing the moderate ancestral diversity scenario in which both Europeans and Asians shared the same causal variant, against the setting in which they had different causal variants in low LD with one another. In the moderate diversity setting, power losses from the presence of allelic heterogeneity in half of the samples ranged from 10% in perfect data up to 23% in imputed data, irrespective of MAF (Table 3); these same losses are observed between single ancestry and allelic heterogeneity in the moderate diversity setting when the data are perfect and at MAFs 10 and 20% in imputed data. Specifically, in individual regions, perfect data had power losses from allelic heterogeneity between 3.8 and 26.2%, with median 12.3%. For imputed data, power losses from moderate diversity with heterogeneity over that without heterogeneity (or single ancestry) are between 5.7% (5.6%) and 33.2% (33.2%) with median 16.4% (16.2%). At MAF 5%, in imputed data, the power for the single ancestry setting is on average 2.6% lower than that of the setting in which there is no heterogeneity in the moderate diversity setting. Upon repeating the meta-analysis with random effects for *IGF2BP2* in the presence of allelic heterogeneity and for perfect data, there was a larger power reduction; fixed-effects meta-analyses yielded powers of 0.819, 0.886, and 0.865 at respective MAFs of 5, 10, and 20%, whereas the coinciding powers from random-effects meta-analyses were 0.73, 0.795, and 0.51.

In the locus heterogeneity scenario, we found that null cohorts (East Asian, African) had a dramatic impact on the power to detect the causal variant that was associated in only two (European) of the six cohorts (Table 4). In both perfect and imputed data, the average power loss over the regions was ∼50%, irrespective of the MAF. The individual region power reductions for perfect data were between 45.7 and 67.9%, with median 60.6%, whereas imputed data had power losses between 52.5 and 75.9%, with median 60.3%. Slightly larger power losses were observed when there was both locus and allelic heterogeneity (different causal variants for European and East Asian, African null cohort), as detailed in Table 4. These results are comparable to previous results^10^ where a fixed-effects analysis of all populations attained a power of 99.7%, whereas the power dropped by 69.3% when African and East Asian samples were null strata. The power losses from the inclusion of samples with a null association at the variant suggest that locus heterogeneity may have a more profound effect on power compared with allelic heterogeneity. Similar power losses are observed in comparing the single ancestry scenario with any of the heterogeneity settings in the high diversity scenario, as there are negligible differences between the powers of the single ancestry and high diversity ancestral composition in the absence of heterogeneity.

The manner in which the credible set is constructed makes the assumption of a common causal variant that does not hold in the heterogeneity settings, such that a low fine-mapping resolution is expected in these settings. As an illustration, credible sets were constructed when there is moderate diversity and allelic heterogeneity in *IGF2BP2*, as well as both allelic and locus heterogeneity in the high diversity scenario. Presence of the signal in only one group (Eu) resulted in very large 95% credible sets, consisting of at least 1500 variants (results not provided). Use of random effects rather than fixed effects reduced the size of the credible sets by ∼200 SNPs, but credible sets were constructed in <10% of the replications; few replications had a SNP that passed the genome-wide significance threshold.

## Discussion

There is a clear improvement in fine-mapping resolution when synthesising data across highly diverse samples (European/Asian/African) in comparison with the analyses of samples from the same ancestry (European). A clear trend of increased fine-mapping resolution is not apparent in comparisons between common ancestry meta-analyses and those consisting of samples of European and Asian ancestry. This may be because of individuals of European and Asian ancestry being more closely related with each other than with those of African ancestry, as had been illustrated in previous relatedness analyses.^9^ These results indicate that the meta-analysis of ancestrally diverse samples results in an improvement in fine-mapping resolution, provided that the composition includes those of African ancestry. For further investigation of this improvement, we examined African-only meta-analyses that displayed a high fine-mapping resolution, suggesting that increasing the sample size of African ancestry samples is more likely to improve fine-mapping resolution than increasing sample diversity. It would also be of interest to investigate the inclusion of Hispanic cohorts, given the current efforts in establishing large Hispanic cohorts, the admixing history, and ethnic diversity within such cohorts. Given the fine-mapping improvements observed from the inclusion of African ancestry samples, and that the LD levels in Hispanic samples tend to fall between those of Africans and Europeans/East Asian, inclusion of Hispanic samples is expected to contribute to refinement in fine-mapping over that of European and East Asian samples.

Methods for trans-ethnic meta-analyses have been compared in extensive simulation studies,^10^ yet the impact of ancestral composition in study design has not previously been investigated. Although the conducted simulation studies are limited by taking place under an ideal scenario in which imputation reference panels consist of populations that are exact matches for the study samples, they act as a gauge for the performance of the various ancestral composition study designs. This study highlights the benefits of trans-ethnic meta-analysis for detecting novel complex trait loci and fine-mapping causal variants, when there is a shared causal variant. It also considers the effect on power from the inclusion of cohorts where the variant is null that may occur if it was discovered in African populations and did not segregate outside of Africa, or vice versa. In this case, the fixed-effects (and random-effects) meta-analysis is exposed to a dramatic power loss when the sample size is fixed over the different ancestral diversity scenarios. Therefore, when there is a restriction on sample size (eg, cost), careful consideration is needed in designing the ancestral composition for the meta-analysis, depending on the assumption of shared causal variants or locus heterogeneity.

Meta-analyses are often performed with an intention of maximising the number of available cohorts, with or without African ancestry cohorts. This is often owing to the restricted opportunities to include African cohorts, either because of GWAS data not existing or not readily available for sharing and for meta-analysis. Our simulation results indicate the importance of including African ancestry samples in fine-mapping studies, and that high gains in resolution are possible with their inclusion. Considering the high resolution in the African-only analyses, it may be beneficial to include a few small African cohorts (eg, size 1000 each) rather than an additional couple of large European cohorts (eg, size >10 000). The possibility of deriving benefits that have a global impact may therefore be more likely by developing the capacity for genomic sciences in Africa, such that an increased number of African-focussed GWASs may be performed. This includes enabling and empowering more African researchers to carry out such studies in their populations.

## Figures and Tables

**Table 1:** Summary measure (and SE) over the five regions of the probability that the causal variant is in the 95% credible set

*Ancestry combination*	*Data type*	*MAF 5%*	*MAF 10%*	*MAF 20%*
Single (Eu)	Perfect	0.944 (0.00367)	0.894 (0.00447)	0.929 (0.00426)
	Imputed	0.864 (0.00768)	0.815 (0.00650)	0.892 (0.00562)
Moderate (Eu/EA)	Perfect	0.922 (0.00558)	0.903 (0.00442)	0.936 (0.00410)
	Imputed	0.894 (0.00644)	0.846 (0.00567)	0.920 (0.00490)
High (Eu/Ea/Af)	Perfect	0.930 (0.00395)	0.910 (0.00421)	0.928 (0.00440)
	Imputed	0.892 (0.00517)	0.845 (0.00559)	0.853 (0.00618)
Single (Af)	Perfect	0.941 (0.00358)	0.929 (0.00404)	0.921 (0.00541)

Perfect and imputed data results are given in the upper and lower portion of each cell, respectively. Credible sets were constructed only when the lead SNP had *P*-value below 5E−08.

**Table 2:** Summary measure of the medians over the five regions for the regional median number of SNPs in the 95% credible sets

*Ancestry combination*	*Data type*	*MAF 5%*	*MAF 10%*	*MAF 20%*
Single (Eu)	Perfect	4.6	2.0	7.8
	Imputed	8.2	3.6	9.0
Moderate (Eu/EA)	Perfect	3.8	3.0	10.8
	Imputed	4.2	3.0	11.6
High (Eu/Ea/Af)	Perfect	1.8	1.0	3.0
	Imputed	2.4	1.6	4.6
Single (Af)	Perfect	1.0	1.0	1.6

Perfect and imputed data results are given in the upper and lower portion of each cell, respectively. Credible sets were constructed only when the lead SNP had *P*-value below 5E−08.

**Table 3:** Over the five regions, the mean power to detect the variant that is causal in the European samples c_1_ (at significance level 0.05) in the absence/presence of allelic heterogeneity; positive detection occurs when the *P*-value for the single variant test at c_1_ is below 0.05

*Sample composition and causal variants*	*Data type*	*MAF 5%*	*MAF 10%*	*MAF 20%*
Eu-c_1_ (single ancestry)	Perfect	1.000	1.000	1.000
	Imputed	0.970	0.999	0.999
Eu/EA-c_1_ (no heterogeneity)	Perfect	1.000	1.000	1.000
	Imputed	0.996	1.000	0.999
Eu-c_1_, EA-c_2_ (allelic heterogeneity)	Perfect	0.892	0.870	0.832
	Imputed	0.862	0.829	0.775

Perfect and imputed data results are given in the upper and lower portion of each cell, respectively. The sample compositon for each meta-analysis includes European (Eu) and East Asian (EA) whereas causal variants are denoted by c_1_, and c_2_, respectively, for association at c_1_ and c_2_.

**Table 4:** Summary measure over the five regions of the power to detect the variant that is causal in the European samples c_1_ (at significance level 0.05) in the presence of allelic and locus heterogeneity; positive detection occurs when the *P*-value for the single variant test at c_1_ is below 0.05

*Sample composition and causal variants*	*Data type*	*MAF 5%*	*MAF 10%*	*MAF 20%*
Eu-c_1_ (single ancestry)	Perfect	1.000	1.000	1.000
	Imputed	0.970	0.999	0.999
Eu/EA/Af-c_1_ (no heterogeneity)	Perfect	1.000	1.000	1.000
	Imputed	0.998	1.000	0.999
Eu-c_1_, EA-c_2_, Af-0 (allelic and locus heterogeneity)	Perfect Imputed	0.419 0.257	0.494 0.426	0.412 0.359
Eu-c_1_, EA/Af-0 (locus heterogeneity)	Perfect	0.413	0.539	0.432
	Imputed	0.320	0.495	0.399

Perfect and imputed data results are given in the upper and lower portion of each cell, respectively. The sample compositon for each meta-analysis includes European (Eu), East Asian (EA), and African (Af), whereas causal variants are denoted by 0, c_1_, and c_2_, respectively, for null data, association at c_1_, and association at c_2_.
