# Supplementary material for: Trans-ethnic study design approaches for fine-mapping
Source: Eur J Hum Genet. 2016 Feb 3;24(9):1330–6. doi: 10.1038/ejhg.2016.1 (PMC4856879; doi:10.1038/ejhg.2016.1)
Supplement: Supplementary Table S1 [file ejhg20161x1.docx]

Supplementary Table S1: Individual locus results for the median number of SNPs in 95% credible sets. Each sample has causal variant c_1_ with MAF specified in the European populations. Credible sets were constructed only when the lead SNP had p-value below 5E-08. Perfect and imputed data results are given in the upper and lower portion of each cell, respectively.

| Ancestry  Combination\Locus | | | | *IGF2BP2* | *CDKAL1* | *CDKN2A/B* | *KCNQ1* | *FTO* |
| --- | --- | --- | --- | --- | --- | --- | --- | --- |
| MAF 5% in Eu | | | | | | | | |
| Single  (Eu) | | | Perfect  Imputed | 2.0  8.0 | 7.0  10.0 | 1.0  1.0 | 1.0  4.0 | 12.0  18.0 |
| Moderate  (Eu/EA) | | | Perfect  Imputed | 6.0  6.0 | 4.0  4.0 | 1.0  1.0 | 1.0  1.0 | 7.0  9.0 |
| High  (Eu/EA/Af) | | | Perfect  Imputed | 1.0  1.0 | 1.0  3.0 | 1.0  1.0 | 1.0  1.0 | 5.0  6.0 |
| Single (Af) | | | Perfect | 1.0 | 1.0 | 1.0 | 1.0 | 1.0 |
| MAF 10% in Eu | | | | | | | | |
| Single  (Eu) | | Perfect  Imputed | | 1.0  2.0 | 3.0  4.0 | 1.0  2.0 | 2.0  6.0 | 3.0  4.0 |
| Moderate  (Eu/EA) | | Perfect  Imputed | | 2.0  2.0 | 8.0  8.0 | 3.0  3.0 | 1.0  1.0 | 1.0  1.0 |
| High  (Eu/EA/Af) | | Perfect  Imputed | | 1.0  1.0 | 1.0  1.0 | 1.0  1.0 | 1.0  3.0 | 1.0  2.0 |
| Single (Af) | | Perfect | | 1.0 | 1.0 | 1.0 | 1.0 | 1.0 |
| MAF 20% in Eu | | | | | | | | |
| Single  (Eu) | Perfect  Imputed | | | 4.0  7.0 | 7.0  7.0 | 13.0  14.0 | 3.0  4.0 | 12.0  13.0 |
| Moderate  (Eu/EA) | Perfect  Imputed | | | 9.0  9.0 | 35.0^*^  36.0^*^ | 1.0  1.0 | 2.0  3.0 | 7.0  9.0 |
| High  (Eu/EA/Af) | Perfect  Imputed | | | 1.0  3.0 | 6.0  7.0 | 4.0  5.0 | 2.0  4.0 | 2.0  4.0 |
| Single (Af) | Perfect | | | 1.0 | 1.0 | 2.0 | 2.0 | 2.0 |

^*^When the causal variant had MAF 20% in *CDKAL1,* the median was noticeably larger for the moderate diversity setting over any other. In this locus, we found that the causal SNP was the lead SNP with low probability. In addition, there was a tendency for many SNPs to have similarly small p-values close to genome-wide significance, and thus large ABFs. In turn, the posterior probabilities were much smaller, so that a large set was needed to reach a 95% credible set.
