# Supplementary material for: Trans-ethnic study design approaches for fine-mapping
Source: Eur J Hum Genet. 2016 Feb 3;24(9):1330–6. doi: 10.1038/ejhg.2016.1 (PMC4856879; doi:10.1038/ejhg.2016.1)
Supplement: Supplementary Table S2 [file ejhg20161x2.docx]

Supplementary Table S2: Resulting p-values from each paired t-test of medians between ancestral groups within each gene. The column headings indicate the MAF of the shared causal variant in the European populations, while the rows indicate the direction of the test for the difference in medians. Perfect and imputed data results are given in the upper and lower portion of each cell, respectively.

| Ancestry  Combination | Data  Type | MAF  5% | MAF  10% | MAF  20% |
| --- | --- | --- | --- | --- |
| Single > Moderate | Perfect  Imputed | 0.31  0.032 | 0.77  0.36 | 0.66  0.63 |
| Moderate > High | Perfect  Imputed | 0.051  0.069 | 0.10  0.21 | 0.12  0.15 |
| Single > High | Perfect  Imputed | 0.071  0.023 | 0.045  0.0055 | 0.035  0.047 |
