# Supplementary material for: Trans-ethnic study design approaches for fine-mapping
Source: Eur J Hum Genet. 2016 Feb 3;24(9):1330–6. doi: 10.1038/ejhg.2016.1 (PMC4856879; doi:10.1038/ejhg.2016.1)
Supplement: Supplementary Table S3 [file ejhg20161x3.docx]

Supplementary Table 3: Median MAF of causal variants in the East Asian and African samples, where the MAF was specified as 5%, 10% or 20% in Europeans.

| Locus\  Population | *IGF2BP2* | CDKAL1 | CDKN2A/B | KCNQ1 | FTO |
| --- | --- | --- | --- | --- | --- |
|  | MAF 5% in Eu | | | | |
| East Asian | 0.02 | 0.18 | 0.08 | 0.23 | 0.13 |
| African | 0.07 | 0.07 | 0.09 | 0.08 | 0.13 |
|  | MAF 10% in Eu | | | | |
| East Asian | 0.3 | 0.18 | 0.08 | 0.11 | 0.1 |
| African | 0.12 | 0.11 | 0.27 | 0.12 | 0.18 |
|  | MAF 20% in Eu | | | | |
| East Asian | 0.2 | 0.44 | 0.43 | 0.23 | 0.3 |
| African | 0.45 | 0.08 | 0.18 | 0.17 | 0.3 |
